# Does Heart Rate Variability Biofeedback Enhance Executive Functions Across the Lifespan? A Systematic Review

**DOI:** 10.1007/s41465-021-00218-3

**Published:** 2021-06-30

**Authors:** Doriana Tinello, Matthias Kliegel, Sascha Zuber

**Affiliations:** 1 Department of Psychology, Faculty of Psychology and Sciences of Education, University of Geneva, 28 Boulevard du Pont d’Arve, 1205 Geneva, Switzerland; 2 Swiss National Centre of Competence in Research LIVES Overcoming Vulnerability: Life Course Perspectives, Lausanne and Geneva, Geneva, Switzerland; 3 Centre for the Interdisciplinary Study of Gerontology and Vulnerability, University of Geneva, Geneva, Switzerland

**Keywords:** Heart rate variability, Biofeedback, Executive functions, Systematic review, Cognitive enhancement, Intervention

## Abstract

The scope of this systematic review was to summarize the existing literature on the effects of heart rate variability biofeedback (HRV-BF) on executive functions (EFs) across the lifespan. Specifically, it aimed to investigate the factors that may affect the efficacy of HRV-BF interventions, such as the study population, duration and intensity of the intervention, or the technical equipment. This review was conducted according to the Preferred Reporting Items for Systematic Reviews and Meta-Analyses (PRISMA) guidelines. Studies that measured and presented at least one EF were included. We included controlled and uncontrolled trials involving clinical and general populations. From the initial list of 137 papers, 16 final studies were reviewed, with 777 participants. Fifty-six percent of the studies included in this review reported significant positive effects of HRV-BF intervention on at least one EF. Attention was the domain that most often benefited from the intervention. The majority of EF improvements (78%) occurred in studies that addressed patient populations or individuals that may present particular profiles: individuals exposed to stress, professional athletes, war veterans, children and adults with ADHD, and clinical older patients. The remaining studies (22%) that reported significant improvements focused on the general population. Efficacy was neither related to the duration or intensity of the intervention nor related to the technical equipment. Overall, our review shows that HRV-BF may be beneficial (a) to increase attentional skills, inhibition, and working memory and (b) when targeting more vulnerable individuals or individuals with particular profiles. However, further development of standardized, controlled protocols and consistent reporting of effect sizes may contribute to establishing the relevance of HRV-BF biofeedback interventions within the field of cognitive enhancement.

**Supplementary Information:**

The online version contains supplementary material available at 10.1007/s41465-021-00218-3.

## Introduction

In recent years, training interventions aimed to maintain or improve executive functions (EFs) have received considerable attention, helping general and vulnerable populations develop or preserve an optimal level of functioning (Diamond & Ling, 2016). In this context, cognitive training is the most used approach to enhance EFs, although it is still unclear to what extent the training-induced benefits generalize beyond the trained tasks (Harvey et al., 2018). Recently, heart rate variability biofeedback (HRV-BF) has received growing interest because of its effectiveness in modifying heart rate variability and its positive influence on emotions, cognitive functioning, and physical wellbeing (Lehrer et al., 2020). However, today, only few studies have explored the potential impact of HRV-BF on *enhancing* cognitive functioning. This systematic review aims to summarize available findings and identify factors that may affect the efficacy of HRV-BF on EFs across healthy and clinical populations of different ages.

### What Is Heart Rate Variability and How Is It Measured?

The term *heart rate variability* (HRV) refers to the changes in the time interval between two consecutive heartbeat (Shaffer & Ginsberg, 2017). Considered as a marker of an organism’s adaptability and resilience, HRV reflects the balance of the autonomous nervous system (Shaffer et al., 2014). While too much variability in heart rate can be harmful to efficient physiological functioning, too little variability correlates with a reduced capacity to adapt to environmental demands and stressors (Shaffer et al., 2014).

There are three widely used approaches to measure HRV: time-domain, frequency-domain, and non-linear measures (Shaffer & Ginsberg, 2017). *Time-domain* measures estimate the amount of variability in time intervals between successive interbeats. The most common time-domain measures are as follows: the standard deviation of all normal NN intervals^1^ (SDNN), the standard deviation of the average NN intervals for each 5 min segment of 24-h HRV recordings (SDANN), the proportion of successive RR^2^ intervals that are > 50 ms apart (pNN50), and the root mean square of successive RR interval differences (RMSSD) (Shaffer & Ginsberg, 2017).

*Frequency-domain* measures estimate the distribution of the absolute or relative power (energy) into four frequency bands (Shaffer & Ginsberg, 2017). High-frequency (HF) bands (0.15 to 0.4 Hz) are mainly affected by respiratory rhythms from 9 to 24 breaths per min (bpm) and reflect parasympathetic activity (Shaffer & Ginsberg, 2017). Low-frequency (LF) bands (0.04 to 0.15 Hz) are affected by baroreceptor activity and by respiratory rhythms from ~ 3 to 9 bpm; they reflect both sympathetic and parasympathetic activities. Very low-frequency (VLF) and ultra-low-frequency (ULF) (VLF, 0.0033 to 0.04 Hz; ULF, ≤ 0.0033 Hz) bands are spectral components with very low oscillations; today, there is no consensus on their origin, with studies suggesting that variations in VLF and ULF are under the influence of hormones, temperature regulation, and/or physical activity (Sztajzel, 2004). While the VLF has been found to be a marker of sympathetic activity, the ULF might reflect circadian and neuroendocrine rhythms (also see Akselrod et al., 1981; Serrador et al., 1999; Sztajzel, 2004). In addition to these four main frequencies, frequency-domain measures also estimate the ratio of LF to HF, which reflects the ratio between sympathetic and parasympathetic activities (Shaffer & Ginsberg, 2017; Stein et al., 1994); the HRV coherence ratio, which is represented by a high-amplitude peak in the LF region (typically around 0.1 Hz) of the power spectrum with no major peaks in the other bands and reflects a state of appreciation or compassion associated with a more coherent rhythm (McCraty, 2017); the absolute power, which indicates the signal energy found within a frequency band; the relative power, which is estimated by the percentage of total power in each frequency band; and the total power, which is the sum of the energy in all frequency bands (Shaffer & Ginsberg, 2017; Stein et al., 1994).

Finally, *non-linear measurements* of HRV try to quantify the non-linear relationship between RR intervals (see Shaffer & Ginsberg, 2017, for details).

### Why Is HRV Important?

Extensive literature associates HRV with physiological health, emotions, and the level of cognitive functioning (Thayer & Lane, 2000; Thayer et al., 2009; Winkelmann et al., 2017). For example, research suggests that individuals with higher HRV are better at reducing stress and negative emotions (Geisler et al., 2013), more self-aware, and also better at understanding the mind of others (Lischke et al., 2017). In contrast, lower HRV has been observed in patients with reduced cardiac regulatory capacity (Berntson et al., 2008) and has been associated with obsessive–compulsive drinking behavior (Quintana et al., 2013), inflammatory markers, hypertension, and depression (Lampert et al., 2008). Further, HRV has been postulated to influence cognitive functioning across the lifespan. For example, higher HRV predicted better performance on a working memory task in children (Staton et al., 2009) and better working memory and processing speed in adults (Hansen et al., 2003). In contrast, lower HRV in old age was related to worse performance on inhibitory tasks and reduced processing speed (Mahinrad et al., 2016).

Based on the central role of HRV, pioneer studies in the field found that HRV can be voluntarily altered, which was initially achieved through a technique called *respiratory sinus arrhythmia biofeedback*, which is based on the beat-to-beat variability of the heart rate that accompanies breathing (Lehrer, Vaschillo, & Vaschillo, 2000). Further research led to the development of HRV biofeedback as a potential intervention method for treating a multitude of physical, emotional, and psychological issues (Lehrer & Gevirtz, 2014).

### HRV Biofeedback Interventions

HRV-BF is a non-invasive intervention technique developed in the early 1990s (Lehrer et al., 2000). In detail, it consists of displaying an individual’s respiratory and heart rate oscillations on a monitor and instructing the individual to breathe at a certain rhythm—generally comprised between 4.5 and 6.5 cycles per min. Breathing at this pace stimulates the cardiovascular system’s resonant properties that produce the largest oscillations in the heart (Lehrer et al., 2013). Consequently, this leads to greater amplitude of the individual’s HRV (meaning that differences between the time intervals between heartbeats become larger). By seeing their heart rate oscillations and how their breathing impacts the oscillations, individuals learn to modify their breathing to their individual resonant frequency, which is the rate at which the cardiovascular system maximizes HRV (Lehrer et al., 2000).

Since its discovery, the literature showed that HRV-BF constitutes a potentially efficacious treatment for several physiological and psychological conditions, such as cardiovascular disease, chronic obstructive pulmonary disease, post-traumatic stress disorder, stress, depression, and fibromyalgia (Wheat & Larkin, 2010). However, there is still a lack of systematic evidence of the impact of HRV-BF on enhancing cognitive functioning, especially on enhancing EFs. Consequently, this systematic review sought to investigate how modifying HRV can influence EFs across the lifespan.

### Definition of Executive Functions

EFs encompass cognitive processes involved in controlling, organizing, and integrating information (Diamond, 2013). On the one hand, EFs are crucial in everyday functioning because they allow planning, reasoning, making decisions, and solving problems. On the other hand, EFs also support everyday lives because they control and regulate emotions (Nguyen et al., 2019). Although there are different approaches to describing key aspects of EFs, today, the most prevalent model defines three core facets: inhibition, working memory, and cognitive flexibility (Friedman & Miyake, 2017). Inhibition represents the ability to control one’s behavior, thoughts, and emotions when they are not appropriate or not needed. Inhibition also involves resisting interfering information when non-relevant for the ongoing situation (Diamond, 2013). Working memory describes the ability to retain and manipulate information over a short time, which is necessary to bind several elements in a global entity or, on the contrary, to dissociate and then reorganize that information differently (Baddeley, 2012). Cognitive flexibility designates the ability to change one’s perspective or one’s approach to a problem in order to adjust to new demands from the changing environment (Diamond, 2013).

EFs play a crucial role across the different phases of lifespan development. In childhood, EFs are associated with academic achievement and predict social functioning in adolescence (Er-Rafiqi et al., 2017). In adulthood, EFs are related to mental and physical health, low productivity or job search difficulties, marital harmony, and public safety (Bailey, 2007; Denson et al., 2011). In old adulthood, EFs strongly contribute to daily functioning and maintaining autonomy (Jefferson et al., 2006).

Given how central EFs are to managing daily tasks across the lifespan, a primary goal for public health research is to develop training interventions that improve EFs, thereby preventing vulnerable populations from falling below a critical threshold of executive functioning. Different approaches have been used to pursue these goals, but cognitive training represents the most used and most examined approach (see Kliegel, Hering, Ihle, & Zuber, 2017). Despite the numerous studies that applied this approach, it is still controversial to what extent such interventions actually benefit individuals (Harvey et al., 2018). The cognitive training literature shows mixed results regarding training gains and transfer effects. Many studies suggest that benefits are limited and remain restricted to the trained tasks without any broader transfer to untrained tasks (for systematic reviews, see Joubert & Chainay, 2018; Nguyen et al., 2019). Consequently, researchers as well as practitioners have been looking for alternative approaches to enhance cognition. In this context, HRV-BF has received growing interest because of its effectiveness in altering HRV and positively influencing mental and physical wellbeing (Lehrer et al., 2020).

### The Relationship Between HRV and EFs

The relation between HRV and EFs has been well documented in the works of Thayer et al. (2009). These authors have proposed a conceptual framework, the neurovisceral integration model, in which HRV and EFs share common neural bases. This model is based on the central autonomic network, which comprises cortical and subcortical brain regions connected to the heart through sympathetic and parasympathetic innervations (Benarroch, 1993). The neurovisceral integration model supports evidence that prefrontal cortical activity can influence cardiovascular functioning and that dysregulation of HRV can negatively impact EFs (Thayer et al., 2009). According to Thayer et al. (2009), this complex neural network plays a crucial role in influencing our ability to cope with rapidly changing environments, perform goal-directed behaviors, and select, maintain, update, and inhibit information, which are all aspects related to EFs. Because of the association between HRV and EFs, a certain number of studies have investigated the effectiveness of HRV-BF interventions in ameliorating EF outcomes. However, these studies significantly differ in terms of the target population, the technical equipment that was used, the intervention protocol, and the duration and intensity of the intervention. Consequently, the existing literature provides mixed results, making it unclear whether HRV-BF benefits EFs or not. Further, it is unclear whether potential benefits could depend on specific factors and conditions of the intervention. With the present review, we aimed to summarize the studies investigating the effects of HRV biofeedback training on EFs across the lifespan. Further, we aimed to examine differences in protocols and equipment across the studies and to identify factors that may influence the efficacy of HRV biofeedback interventions. Finally, we aimed to provide an outline of open questions and suggestions for future research.

## Method

This systematic review was conducted according to the Preferred Reporting Items for Systematic Reviews and Meta-Analyses (PRISMA) statement guidelines (Moher et al., 2015).

**Fig. 1:**
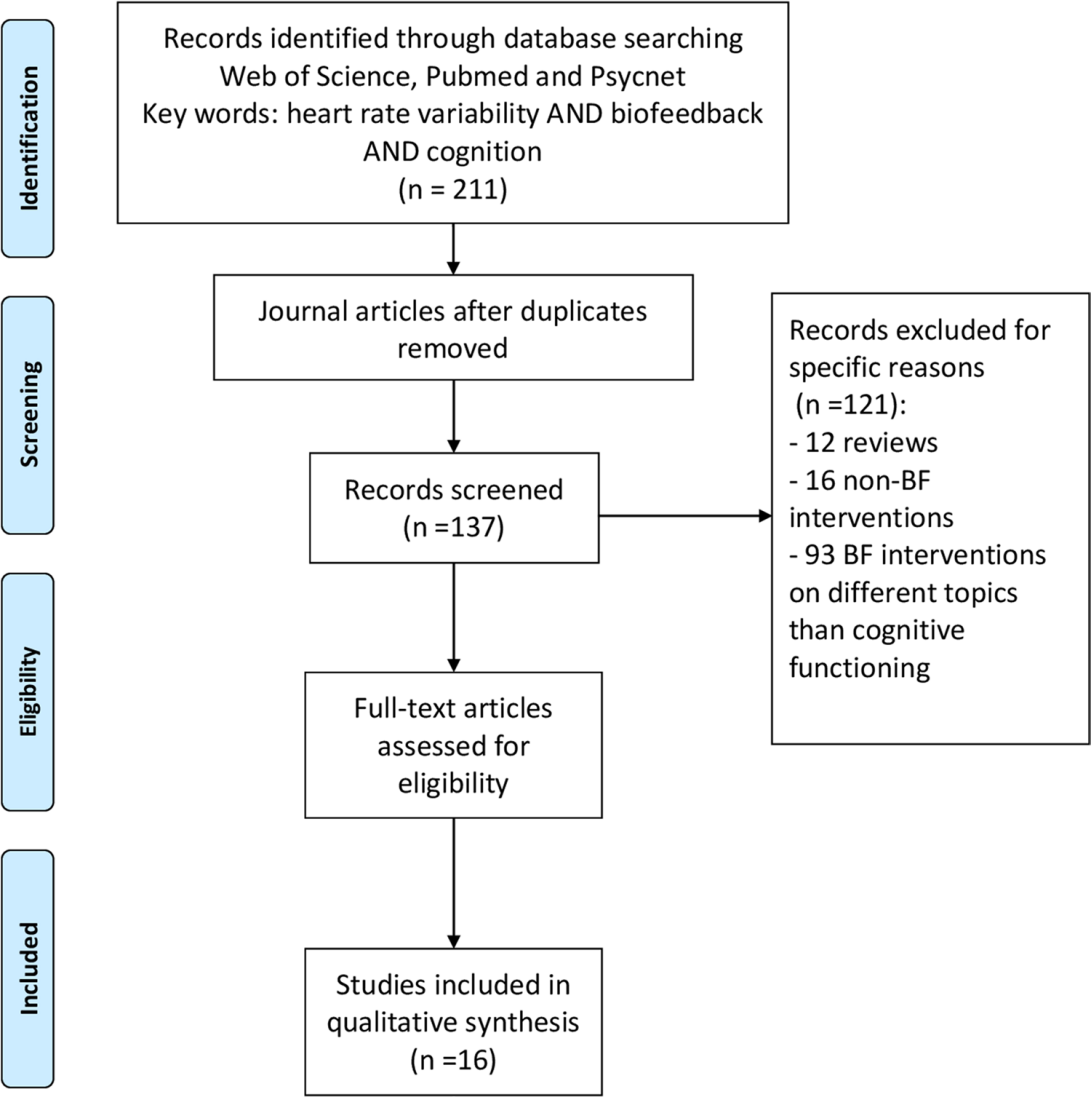
Flow diagram of study selection for systematic review of published research on HRV-BF intervention on EFs

To identify the relevant studies for this literature review, we searched Web of Science, PubMed, and PsycNet for articles published until March 2020. The search terms included the following sets of keywords: (heart rate variability) AND (biofeedback) AND (cognition OR cognitive performance OR attention OR attentional control OR controlled attention OR executive control OR executive functions OR executive function OR executive functioning OR frontal lobe OR frontal lobes OR frontal function OR inhibition OR inhibitory control OR working memory OR updating OR shifting OR switching). We limited the search to journal articles only. Articles were considered eligible for this literature review (1) if they described the HRV-BF intervention, (2) if they incorporated a pre–post assessment, and (3) if they assessed at least one EF or a related construct as an outcome measure: attention and academic achievement. Although the relationship between attention and EFs is still debated, there seems to be a consensus on the fact that EF appears as a unique dimension early in life and develops into a multidimensional construct later in adulthood (Klenberg et al., 2010; Mccabe et al., 2010). Thus, this review accepted studies that examined attention as a unique component. Furthermore, we included one study that indirectly assessed EFs through academic achievement. The reason for doing this is that there is evidence for the association between EFs and achievement test performance. For example, performance on inhibition and working memory tasks relates to performance in mathematics and reading (Best et al., 2011; Blair & Diamond, 2008). (4) Only studies with empirical data were included; meta-analyses, reviews, or theoretical contributions were excluded. The initial search generated 211 papers reduced to 137 after the removal of duplicates. Of these 137 papers, 12 were reviews and thus excluded. Sixteen articles reported correlational results between HRV parameters and emotional, cognitive, and physical outcomes but did not include any biofeedback (BF) intervention. Thus, these articles were excluded as well. Ninety-three articles used HRV-BF but did not examine EFs and were therefore also excluded. The final selection included 16 articles (see Fig. 1 for a flow diagram).

For each of the selected studies, the main characteristics were extracted: year of publication; target population; sample size; sex; age of participants; study focus; study design; type of the biofeedback intervention; whether there was a control condition, duration, and frequency of intervention sessions; cognitive measurements; EF outcomes; HRV measurements; HRV outcomes; and the equipment that was used (see Tables 1, 2, and 3). With the aim of reporting consistent indicators across all studies, we selected studies that assessed common and comparable EF outcomes. This left 11 of the 16 studies (de Bruin et al., 2016; Groeneveld et al., 2019; Jester et al., 2019; Kenien, 2015; Lee & Finkelstein, 2015; May et al., 2019; Prinsloo et al., 2011; Rusciano et al., 2017; Schumann et al., 2019; Sherlin et al., 2010; Sutarto et al., 2013). Of the five excluded studies, one study was excluded because it was unique in measuring academic performance as an indicator of EFs (Bradley et al., 2010). Four studies were excluded because the reported data were insufficient to calculate effect sizes (Ginsberg et al., 2010; Kim et al., 2013; Pop-Jordanova & Chakalaroska, 2008; Raaijmakers et al., 2013). When we examined the 11 remaining studies, we realized that they largely differed in the reporting of effect size (Cohen’s *d* vs. partial eta squared vs. no reported effect sizes) and on which comparison the effect sizes were based on (pre–post comparison of the intervention group only vs. pre–post control group designs). Thus, we calculated the effect sizes for the eight independent-groups studies based on Morris (2008)’s formula numbers 8 to 10 for pre-test–post-test controlled studies, using the pooled pre-test standard deviation. When studies included two comparative groups (de Bruin et al., 2016; May et al., 2019), each comparison was considered as a separate study. We also calculated the effect sizes for one single-group study applying Cohen’s formula number 9 for repeated measures in within-subjects designs (Lakens, 2013). For a summary of the characteristics of the studies evaluated, see Tables 1, 2, and 3.

**Table 1:** Study features

Study	Type of sample	Sample size (*n* of women) (Exp/Ctrl)	Age^1^	Study focus	Study design	Control condition
Bradley et al. (2010)	High school students	136 (53%) 77/59	Exp: 15.3 (0.44) Ctrl: 15.3 (0.44)	Effects of TestEdge program on stress and anxiety management	Quasi-experimental field design/pre–post assessment	Waiting list
de Bruin et al. (2016)	Young highly stressed adults	75 (55) MM: 27 (20) HRV-BF: 25 (17) PE: 23 (18)	MM: 26.32 (5.03) HRV-BF: 26.99 (6.53) PE: 25.28 (4.42)	Effect of 3 different interventions on attention control, executive functioning, mindful awareness, self-compassion, and worrying	Comparative groups Pre–post assessment	MM PE
Ginsberg et al. (2010)	War veterans	10 5/5	Outpatient clinic: 29.4 (2.5) Active soldiers: 34.2 (2.6)	Effect of HRV-BF intervention on combat veterans	Pilot study Pre–post assessment	Veterans PTSD^2^
Groeneveld et al. (2019)	ADHD patients	139 adults: 39 (27) Children: 100 (28)	Adults: 32.1 (11.6) Children: 10.6 (2.9)	Impact of the combination of NF^3^ and HRV-BF on ADHD symptoms	Pre–post assessment	N/A^4^
Jester et al. (2019)	Older adults	18 (14)	78.15 (9.18)	Cognitive and psychiatric effects of HRV biofeedback on older adults	Pre–post assessment	N/A
Kenien (2015)	Children with emotional disturbances	63 (13%)	Range: 7–14	Impact of heart coherence on executive functions in children with emotional disturbances	Quasi-experimental field design/pre–post assessment	Active
Kim et al. (2013)	Severe brain injury patients	13 (6)	Median age: 44 (range: 23–63)	Response to HRV-BF intervention on executive functioning and brain injury	Pilot study, single-treatment, non-randomized, unblinded quasi-experimental design with repeated measures	N/A
Lee and Finkelstein (2015)	Healthy adults	14 (4)	36.64 (6.85)	Effect of StressEraser on stress and cognitive performance	Cross-over study	N/A
May et al. (2019)	College students	90 (82%) HIIT: 30 HRVCB: 30 Ctrl: 30	18.55 (0.99)	BF intervention to reduce school burnout and improve cardiac functioning in college students	Comparative groups design Pre–post assessment	HIIT Waiting list
Pop-Jordanova and Chakalaroska (2008)	High school students	50 EEG-PAT: 30 EDR: 10 HRV-BF^5^: 10	Range: 16–18	Comparison of 3 biofeedback modalities for better achievement in high school students	Comparative groups Pre–post assessment	EEG-PAT EDR
Prinsloo et al. (2011)	Senior manager exposed to work-related stress with high perceived stress	18 9/9	Exp: 33 (± 6) Ctrl: 34 (± 6)	Effect of 10-min HRV-BF on cognitive performance and affect score during induced stress	Randomized controlled trial Pre–post assessment	Active
Raaijmakers et al. (2013)	University students	28 16/12	22 (range: 19–27)	Impact of HRV and skin conductance BF on physiological, affective, and cognitive variables	Triple blind randomized control Pre–post assessment	Sham control
Rusciano et al. (2017)	Professional football players	20 10/10	Exp: 30.0 (3.8) Ctrl: 30.7 (4.3)	Impact of BF intervention on attention, resilience, and injury prevention	Single-blind Pre–post assessment	Active
Schumann et al. (2019)	Healthy adults	24 14 (7)/10 (5)	Exp: 30 ± 9 years (range: 22–52) Ctrl: 30 ± 13 years (range: 18–55)	Effect of an 8-week HRV-BF intervention on autonomic function and impulsivity	Pre–post assessment	Active
Sherlin et al. (2010)	Healthy adults	43 (48.8%)	33.2 (8.77)	Effects of respiratory sinus arrhythmia (RSA) biofeedback on stress	Pre–post assessment	Concentrative relaxation group
Sutarto et al. (2013)	Healthy female operators	36 19/17	Exp: 35.6 (10.58)	Effect of HRV-BF training on female operator’s cognitive performance	Pre–post assessment	Passive

*Exp* experimental group, *Ctrl* control group, *MM* mindfulness meditation group, *HRV-BF* heart rate variability BF group, *PE* physical exercise group, *HIIT* high-intensity interval training group, *HRVCB* heart rate variability coherence biofeedback group, *EEG-PAT* EEG-peak achievement trainer, *EDR* electrodermal response

^1^Where available, mean (or median) age and standard deviations (in parentheses) per group are specified

^2^Without post-traumatic stress disorder symptoms

^3^Neurofeedback

^4^Not assessed

^5^Heart rate variability biofeedback

**Table 2:** Cognitive measurements and executive function outcomes

Study	Cognitive measurements	Executive function outcomes	Reported effect sizes	Calculated *d* for PPC^1^ and RM^2^
Bradley et al. (2010)	California High School Exit Exam, California Standard Test	Pre–post Exp vs. Ctrl: ns	Missing	
de Bruin et al. (2016)	Attention (Attention Control Scale) Executive functioning (Behavior Rating Inventory of Executive Function-Adult version, BRIEF-A)	Pre–post: ↑ attention ↑ executive functioning	Cohen’s *d* = 0.16 Cohen’s *d* = 0.19	${d}_{\mathrm{p}\mathrm{p}\mathrm{c}2\mathrm{M}\mathrm{M}}$ ^3^ = − 0.08 ${d}_{\mathrm{p}\mathrm{p}\mathrm{c}2\mathrm{P}\mathrm{E}}$ ^4^ = − 0.22
Ginsberg et al. (2010)	Inhibition (Go-NoGo) Memory (Digit Span: WAIS) Verbal memory (Rey Auditory Verbal Learning Test-RAVLT)	Pre–post: $\downarrow$ commissions on Go-No Go $\uparrow$ working memory	Missing	
Groeneveld et al. (2019)^5^	Attention (Full Scale Response Control Quotient (FRCQ); Full Scale Attention Quotient (FAQ))	Pre–post: FRCQ (adults) ns $\uparrow$ FAQ (adults)	Cohen’s *d* = 0.36 Cohen’s *d* = 0.51	
Groeneveld et al. (2019)	Attention (FRCQ; FAQ)	Pre–post: $\uparrow$ FRCQ (children) FAQ(children) ns	Cohen’s *d* = 0.34 Cohen’s *d* = 0.19	
Jester et al. (2019)	Cognitive flexibility (Trail Making Test A/B) Inhibition (Stroop)	Pre–post: $\uparrow$ TMT/A TMT/B: ns Stroop: ns	Cohen’s *d* = 1 Cohen’s *d* = 0.43 Cohen’s *d* = 0.14	
Kenien (2015)	Executive functioning (BRIEF) Inhibition Working memory Cognitive flexibility	Pre–post Exp: ns Pre–post Ctrl: ns	Missing	${d}_{\mathrm{p}\mathrm{p}\mathrm{c}2}$= − 0.01 ${d}_{\mathrm{p}\mathrm{p}\mathrm{c}2}$ = 0.08 ${d}_{\mathrm{p}\mathrm{p}\mathrm{c}2}$ = − 0.21
Kim et al. (2013)	Attention (FAQ) Problem solving (Halstead Category Test (HCT)) Executive functioning (BRIEF-A)	Pre–post: ns	Missing	
Lee and Finkelstein (2015)	Psychomotor vigilance task (PVT)	Pre–post: $\downarrow$ performance RT		${d}_{\mathrm{r}\mathrm{m}}$= − 0.31
May et al. (2019)	Attention (serial subtraction task)	Pre–post Exp^6^ vs. Ctrl: $\downarrow$ math errors	$\small {\eta }_{\mathrm{p}}^{2}=0.090$	${d}_{\mathrm{p}\mathrm{p}\mathrm{c}2\mathrm{C}\mathrm{T}\mathrm{R}\mathrm{L}}$= − 3.62 ${d}_{\mathrm{p}\mathrm{p}\mathrm{c}2\mathrm{H}\mathrm{I}\mathrm{I}\mathrm{T}}$ ^7^ = − 1.32
Pop-Jordanova and Chakalaroska (2008)	Flexibility (Trail Making Test A/B) Working memory (Wechsler MemoryScale-R) Numbering forward Numbering backward	Pre–post^8^: ns Modest improvement for numbers forward ns	Missing	
Prinsloo et al. (2011)	Inhibition (modified Stroop task)	Pre–post Exp vs. Ctrl: $\uparrow$ performance RT $\downarrow$ errors	Missing	${d}_{\mathrm{p}\mathrm{p}\mathrm{c}2}$= − 0.85
Raaijmakers et al. (2013)	Working memory (N-back) Cognitive flexibility (mental rotation task)	Pre–post Exp: $\uparrow$ performance RT $\downarrow$ errors Pre–post Ctrl: $\uparrow$ performance RT $\downarrow$ errors	Missing	
Rusciano et al. (2017)	Attention (visual search task) Inhibition (Stroop)	Pre–post Exp vs. Ctrl: $\uparrow$ performance RT: target absent Target present ns $\uparrow$ accuracy congruent $\uparrow$ accuracy incongruent	$\small {\eta }_{\mathrm{p}}^{2}=0.45$ $\small {\eta }_{\mathrm{p}}^{2}=0.06$ $\small {\eta }_{\mathrm{p}}^{2}=0.22$ $\small {\eta }_{\mathrm{p}}^{2}=0.40$	${d}_{\mathrm{p}\mathrm{p}\mathrm{c}2.\mathrm{a}\mathrm{b}\mathrm{s}}$= − 1.45 ${d}_{\mathrm{p}\mathrm{p}\mathrm{c}2.\mathrm{p}\mathrm{r}\mathrm{e}\mathrm{s}}$ = − 0.45 ${d}_{\mathrm{p}\mathrm{p}\mathrm{c}2.\mathrm{c}\mathrm{o}\mathrm{n}}$ = 2.52 ${d}_{\mathrm{p}\mathrm{p}\mathrm{c}2.\mathrm{i}\mathrm{n}\mathrm{c}\mathrm{o}\mathrm{n}}$ = 3.09
Schumann et al. (2019)	Impulsivity (stop-signal task)	Pre–post Exp vs. Ctrl: GoRT ns SSRT ns	Missing	${d}_{\mathrm{p}\mathrm{p}\mathrm{c}2}$= − 0.17 ${d}_{\mathrm{p}\mathrm{p}\mathrm{c}2}$ = − 0.97
Sherlin et al. (2010)	Inhibition (Stroop errors; modified Stroop task)	Pre–post Exp vs. Ctrl: ns	Cohen’s *d* = 0.29	${d}_{\mathrm{p}\mathrm{p}\mathrm{c}2}$= − 0.21
Sutarto et al. (2013)	Attention (test d2) Memory (Sternberg memory test) Inhibition (Stroop)	Pre–post Exp vs. Ctrl: $\uparrow$ attention $\uparrow$ memory Pre–post Exp: $\uparrow$ interference score	Missing	${d}_{\mathrm{p}\mathrm{p}\mathrm{c}2}$= 0.68 ${d}_{\mathrm{p}\mathrm{p}\mathrm{c}2}$ = − 0.63 ${d}_{\mathrm{p}\mathrm{p}\mathrm{c}2}$ = 0.33

Arrows show the direction of the outcome measure. Upward arrows show an increase of the outcome measure, and downward arrows show a decrease of the outcome measure. “Pre–post Exp vs. Ctrl” refers to an interaction effect where the experimental group saw greater significant change following the intervention than the control group. “Pre–post” refers to a significant change following the intervention where the experimental group was not opposed to a control group (single group design or different comparison groups). “Pre–post Exp” and “Pre–post Ctrl” refers to a main effect of time for the experimental group following the intervention and for the control group not assigned to the intervention. “Post Exp vs. Ctrl” refers to a significant main effect of the experimental group compared to the control group after the intervention

^1^PPC: *d*_ppc2_ effect sizes for pre-test–post-test control group designs calculated according to Morris (2008)

^2^RM: Cohen’s *d*_rm_ effect sizes for repeated measures for within-subjects designs (Lakens, 2013)

^3^Mindfulness meditation

^4^Physical exercise

^5^This study appears in two entries to separate the EF outcomes of the two subgroups (adults and children)

^6^*p* < .05 HRV-BF vs. control

^7^High-intensity interval training

^8^*p* < .05 HRV-BF pre-test vs. HRV-BF post-test (< .05 EEG-PAT pre-test vs. EEG-PAT post-test for numbers forward and numbers backward; no change for EDR condition)

**Table 3:** Frequency, duration, equipment, HRV measures, and results

Study	Session frequency and duration^1^	BF intervention equipment	HRV parameters	HRV outcomes	Reported effect sizes
Bradley et al. (2010)	2 per week for 5 months	Freeze-Framer/emWave	RR SDRR Ln LF Ln HF LnTP LnCoherence ratio	Pre–post Exp vs. Ctrl: $\uparrow$ RR $\uparrow$ SDRR $\uparrow$ LnLF $\uparrow$ LnHF $\tt \uparrow$ LnTP $\uparrow$ Lncoherence ratio	ES: 0.46 0.64 0.55 0.58 0.64 0.52
de Bruin et al. (2016)	5 weeks	StressEraser	N/A		
Ginsberg et al. (2010)	1 per week for 4 weeks	emWave	LF HF VLF TP Coherence ratio	Pre–post: $\uparrow$ LF ns ns $\uparrow$ TP $\uparrow$ coherence ratio	Missing
Groeneveld et al. (2019)^2^	30 sessions BF + NF (30–40-min sessions)	ProComp Infiniti + BioGraph	VLF LF HF	Pre–post: $\downarrow$ %VLF $\uparrow$ %LF $\downarrow$ %HF	Cohen’s *d* = − 0.57 Cohen’s *d* = 1.32 Cohen’s *d* = − 1.27
Groeneveld et al. (2019)			VLF LF HF	Pre–post: $\downarrow$ %VLF $\uparrow$ %LF $\downarrow$ %HF	Cohen’s *d* = − 0.46 Cohen’s *d* = 0.89 Cohen’s *d* = − 0.72
Jester et al. (2019)	2 per week, for 3 weeks (6 30-min sessions)	emWave	N/A		
Kenien (2015)	12 weeks (12 20-min sessions)	emWave	N/A		
Kim et al. (2013)	10 weeks (10 60-min sessions)	emWave	LF/HF Coherence ratio	Pre–post: $\uparrow$ LF/HF $\uparrow$ coherence ratio	${\eta }_{\mathrm{p}}^{2}=0.452$ ${\eta }_{\mathrm{p}}^{2}=0.390$
Lee and Finkelstein (2015)	2 visits (2 10-min sessions)	StressEraser + Zephyr	RR SDNN RMSSD LF HF LF/HF	Pre–post: ns ns ns ns ns ns	Missing
May et al. (2019)	3 weekly 20-min sessions 4 weeks	emWave	HFnu^3^ LFnu	Pre–post Exp: $\downarrow \mathrm{L}\mathrm{F}\mathrm{n}\mathrm{u}$ Pre–post HIIT: $\downarrow \mathrm{L}\mathrm{F}\mathrm{n}\mathrm{u}$ Pre–post Ctrl: $\downarrow \mathrm{L}\mathrm{F}\mathrm{n}\mathrm{u}$	${\eta }_{\mathrm{p}}^{2}=0.700$ ${\eta }_{\mathrm{p}}^{2}=0.700$ ${\eta }_{\mathrm{p}}^{2}=0.700$
Pop-Jordanova and Chakalaroska (2008)	5 sessions	Freeze-Framer/emWave	Coherence ratio^4^	Pre–post: Missing	
Prinsloo et al. (2011)	1 session (1 10-min session)	BioPac/StressEraser	TP LF	Pre–post Exp vs. Ctrl: $\uparrow \mathrm{T}\mathrm{P}$ $\uparrow \mathrm{L}\mathrm{F}$	Missing
Raaijmakers et al. (2013)	7 sessions within 16 days	Active Two	RMSSD	Pre–post Exp vs. Ctrl: ns	Missing
Rusciano et al. (2017)	15 BF sessions 2 per week (30 min)	NeXus-10 Mark II	LF	Resting pre–post Exp: $\uparrow \mathrm{L}\mathrm{F}$	Cohen’s *d* = 0.89
Schumann et al. (2019)	5 sessions per week (4 home session and 1 lab session) 8 weeks	BioPac/Elite HRV	RMSSD SDNN TP RSA BRS	Pre–post Exp vs. Ctrl: $\uparrow$ RMSSD ns ns ns $\uparrow$ BRS	Missing
Sherlin et al. (2010)	1-day session (15 min)	StressEraser + NeXus-10	N/A		
Sutarto et al. (2013)	5 weeks (30–50 min)	I-330 C2	TP LF	Pre–post Exp vs. Ctrl: TPbase ns TPstr ns TPrec ns $\uparrow$ LFbase $\uparrow$ LFstr $\uparrow$ LFrec	Missing

Arrows show the direction of the outcome measure. Upward arrows show an increase of the HRV parameter measure, and downward arrows show a decrease of the HRV parameter measure. “Pre–post Exp vs. Ctrl” refers to an interaction effect where the experimental group saw greater significant change following the intervention than the control group. “Pre–post” refers to a significant change following the intervention where the experimental group was not opposed to a control group (single group design or different comparison groups). “Pre–post Exp” and “Pre–post Ctrl” refers to a main effect of time for the experimental group following the intervention and for the control group not assigned to the intervention. “Post Exp vs. Ctrl” refers to a significant main effect of the experimental group compared to the control group after the intervention. HFnu and LFnu are expressed in normalized units. Nu is calculated by dividing the power of a given frequency component by the total power from which the power of very low frequencies has been subtracted (i.e., LFnu = LF / (LF + HF)); TP: sum of the energy in all the frequency bands; LF/HF: ratio LF [ms^2^] / HF [ms^2^]; TP/LF base, str, rec: measures referring to baseline, stressor, and recovery phase; Coherence ratio: Peak power / (Total power − Peak power)

*SDNN* standard deviation of NN intervals, *RMSSD* square root of the mean square differences between adjacent NN intervals, *LF* low frequency (0.04–0.15 Hz), *HF* high frequency (0.15–0.4 Hz)

^1^Where available, duration (minute per session in parenthesis) is specified

^2^This study appears in two entries to separate the HRV outcomes of the two subgroups (adults and children)

^3^HF and LF are expressed in normalized units. LFnu = 1 − HFnu, LFnu = LF / (LF + HF). LFnu is considered as an index of cardiac sympathovagal tone, so that a reduction of LFnu is associated to an increase of autonomous nervous system modulation (Sgoifo et al., 2015)

^4^Only measured in the HRV-BF group

## Results

### Overall Efficacy for the Enhancement of EFs

The main focus of this review was to assess changes in EFs following the HRV-BF intervention. Table 2 lists the specific EF domains evaluated in each study, the direction of change for each domain, and the effect sizes of these changes when reported in the paper, or when data was sufficient for calculation or could be obtained from the authors. Note that although they may have been interpreted differently in the original studies, for subsequent sections, we consider effect sizes according to Cohen’s benchmarks: small (*d* = 0.20), medium (*d* = 0.50), and large (*d* = 0.80), and small (*η*^2^ = 0.01), medium (*η*^2^ = 0.06), and large (*η*^2^ = 0.14) (Lakens, 2013).

In detail, nine out of the 16 studies (56%) saw improvements in EF performances after the HRV-BF intervention. Specifically, de Bruin et al. (2016) compared the effects of three self-help interventions—mindfulness meditation, HRV-BF, and physical exercise. They found that the three interventions were equally effective in improving attentional control and EFs. However, in the HRV-BF condition, pre–post effect sizes of change for attentional control and for a global index of EFs were small. Further, between-group pre–post differences revealed that the physical exercise group improved more on attentional control than the BF group, with a small effect size. In Ginsberg et al. (2010)’s pilot study, participants significantly improved performance in inhibition and in working memory after the intervention, but there were insufficient data to calculate the effect sizes. Groeneveld et al. (2019) compared attention and response control in adults and children with ADHD. Adults significantly improved attentional control after treatment, showing a medium effect size of the intervention, while they did not significantly improve in response control, which showed a small effect size. In contrast, children increased their performance in response control with a small to medium effect size, but not in attentional control, which revealed a small effect size. However, this study administered a combined intervention of HRV-BF and neurofeedback, making it challenging to disentangle the effect of each technique. Jester et al. (2019) found a large effect size increase in attentional skills, but a non-significant medium effect size increase in cognitive flexibility, and no changes in inhibition after the intervention. May et al. (2019) compared the effect of HRV-BF training to that of high-intensity interval training and a non-training control condition. Results showed a significant interaction with the BF group improving more in attention, with a medium effect size. Between-group pre–post differences contrasting the BF group to the other two conditions revealed large effect sizes. Pop-Jordanova and Chakalaroska (2008) compared the effects of three biofeedback techniques—HRV-BF, neurofeedback training (EEG-peak achievement training), and electrodermal resistance biofeedback (EDR-BF) training—on cognitive flexibility and short-term memory. The HRV-BF group significantly improved only in short-term memory but not in attention and cognitive flexibility. The available data did not allow calculating effect sizes of the changes after the intervention. Prinsloo et al. (2011) compared the effect of HRV-BF to a comparative intervention on performance on inhibition, which integrated a working memory component. Results showed significantly fewer mistakes in the working memory subtask and an increase in inhibition for the BF group compared to the comparison group. A between-group pre–post intervention large effect size was found for inhibition. However, the effect for working memory was unclear: there were no differences between the groups in responding to words, but the BF group significantly performed better in responding to squares. No effect sizes were available. Rusciano et al. (2017) compared the effect of HRV-BF to a motivation treatment intervention on visual selective attention and inhibition. The BF group significantly performed better under the most difficult target-absent condition, with large effect sizes. Similarly, in both the congruent and incongruent conditions of the inhibition task, the BF group performed significantly better than the motivational group, with large effect sizes. Sutarto et al. (2013) did not find between-group differences neither for interference nor for attentional control after the intervention. However, effect sizes for between-group pre–post changes were small to medium.

In contrast to these nine studies, seven studies (44% of all studies) did not show beneficial effects of the BF intervention on cognitive outcomes (see Table 2 for details). Whereas six of these studies did not show any significant pre–post intervention differences (Bradley et al., 2010; Kenien, 2015; Kim et al., 2013; Raaijmakers et al., 2013; Schumann et al., 2019; Sherlin et al., 2010), one study reported a deterioration of performance in attention (Lee & Finkelstein, 2015), for which we calculated a small to medium negative effect size.

### Population Characteristics

The studies varied in terms of sample characteristics. Across the 16 studies, seven studies included subjects of the general population (Bradley et al., 2010; Lee & Finkelstein, 2015; May et al., 2019; Pop-Jordanova & Chakalaroska, 2008; Raaijmakers et al., 2013; Schumann et al., 2019; Sherlin et al., 2010). Four studies included patient or vulnerable populations: ADHD patients (Groeneveld et al., 2019), older adults with and without psychiatric disorders (Jester et al., 2019), children with emotional disturbances (Kenien, 2015), and severe brain injury patients (Kim et al., 2013). Five studies selected non-patient subjects with specific characteristics: senior managers (de Bruin et al., 2016), war veterans (Ginsberg et al., 2010), young adults exposed to stress (Prinsloo et al., 2011), professional football players (Rusciano et al., 2017), and female operators exposed to a high level of stress (Sutarto et al., 2013). In terms of sample age, two studies tested children (Groeneveld et al., 2019; Kenien, 2015). Four studies addressed student populations. They included high school students (Bradley et al., 2010; Pop-Jordanova & Chakalaroska, 2008), college students (May et al., 2019), and university students (Raaijmakers et al., 2013). Nine studies involved young adults and adults (de Bruin et al., 2016; Ginsberg et al., 2010; Groeneveld et al., 2019; Lee & Finkelstein, 2015; Prinsloo et al., 2011; Rusciano et al., 2017; Schumann et al., 2019; Sherlin et al., 2010; Sutarto et al., 2013). Two studies included older adults (Jester et al., 2019; Kim et al., 2013).

### Intervention Features: Duration and Intensity

The number of BF sessions varied considerably across the studies. Three studies consisted of one single session (Lee & Finkelstein, 2015; Prinsloo et al., 2011; Sherlin et al., 2010). One study proposed four sessions (Ginsberg et al., 2010). Seven studies delivered between five and 10 sessions (de Bruin et al., 2016; Jester et al., 2019; Kim et al., 2013; Pop-Jordanova & Chakalaroska, 2008; Raaijmakers et al., 2013; Schumann et al., 2019; Sutarto et al., 2013). Four studies proposed between 12 and 30 sessions (Groeneveld et al., 2019; Kenien, 2015; May et al., 2019; Rusciano et al., 2017). One remaining study extended over 5 months without specifying the total number of sessions (Bradley et al., 2010). Across studies, the duration and frequency of the intervention varied as well. While the shortest session lasted 10 min and was administered only once (Prinsloo et al., 2011), the longest session lasted 60 min in a protocol that delivered 10 weekly sessions (Kim et al., 2013). In eight studies, the duration of each session ranged between 15 and 30 min, but their frequency varied considerably: one single session (Lee & Finkelstein, 2015; Sherlin et al., 2010), daily sessions (de Bruin et al., 2016), one session a week (Kenien, 2015), two sessions per week (Jester et al., 2019; Rusciano et al., 2017), three sessions per week over 4 weeks (May et al., 2019), and seven sessions within 16 days (Raaijmakers et al., 2013). In the most comprehensive study (30 weekly sessions), sessions lasted 30 to 40 min (Groeneveld et al., 2019). One further study delivered five weekly 30–50-min sessions. Five studies did not report the session’s duration (Bradley et al., 2010; Ginsberg et al., 2010; Pop-Jordanova & Chakalaroska, 2008; Schumann et al., 2019).

### Biofeedback Intervention Equipment

The sixteen studies varied in the systems used to deliver HRV-BF intervention and display physiological information. Seven studies used the emWave/Freeze-Framer HRV monitoring system to collect, process, and display physiological data (Bradley et al., 2010; Ginsberg et al., 2010; Jester et al., 2019; Kenien, 2015; Kim et al., 2013; May et al., 2019; Pop-Jordanova & Chakalaroska, 2008). Three studies used StressEraser to display HRV (de Bruin et al., 2016; Lee & Finkelstein, 2015; Sherlin et al., 2010). While one of these studies offered a unique home-based intervention (de Bruin et al., 2016), the other two studies used StressEraser in a laboratory setting: Lee and Finkelstein (2015) utilized a Zephyr heartbeat monitor for collecting and processing HR data (BioHarness 3, Zephyr Technology, MD, USA), while Sherlin et al. (2010) used the NeXus-10 physiological monitoring system (Mind Media, B. V., The Netherlands). One study used the NeXus-10 Mark II system with the BioTrace software to collect, process, and present physiological information (Rusciano et al., 2017). Two studies used the BioPac equipment to collect and process physiological data (Prinsloo et al., 2011; Schumann et al., 2019). The first study used StressEraser to feedback physiological information, and the second one used Elite HRV LLC 2017. One study used the I-330 C2 equipment to collect, process, and present physiological information (Sutarto et al., 2013). One study utilized the Active Two system to measure and process physiological information and displayed it through a video game (Raaijmakers et al., 2013). One final study used the ProComp Infiniti system and the BioGraph software to record, collect, and display physiological data (Groeneveld et al., 2019).

### HRV Outcomes

The specific HRV parameters evaluated in each study and pre-test–post-test effect sizes (where available) are displayed in Table 3. Twelve of the 16 studies reported at least one HRV index. The most common HRV indices were LF power, HF power, LF/HF ratio, coherence index, SDNN, and RMSSD. Of these 12 studies, eight reported a significant amelioration in at least one HRV component in the BF group, from pre- to post-treatment or during the intervention (Bradley et al., 2010; Ginsberg et al., 2010; Groeneveld et al., 2019; Kim et al., 2013; Prinsloo et al., 2011; Rusciano et al., 2017; Schumann et al., 2019; Sutarto et al., 2013). Two studies did not report significant differences between pre- and post-intervention (Lee & Finkelstein, 2015; Raaijmakers et al., 2013). In one study, each condition reported improvements in HRV values (May et al., 2019). One study did not report any HRV outcome (Pop-Jordanova & Chakalaroska, 2008). Four studies did not assess any HRV measure (de Bruin et al., 2016; Jester et al., 2019; Kenien, 2015; Sherlin et al., 2010). As shown in Table 3, of the eight studies that found improvements at a physiological level, only four reported effect sizes. There was a strong pattern of pre–post changes in one study, all with medium to medium-to-large effect sizes on different HRV indices (RR, SDRR, LnLF, LnHF, TP, coherence ratio) (Bradley et al., 2010). In a study of Groeneveld et al. (2019), adults and children consistently improved their physiological parameters as reflected by the increase of LF power and by the decrease of VLF and HF power, all with medium to large effect sizes. Large effect sizes also characterized pre–post changes in LF/HF and coherence ratio in the study of Kim et al. (2013). Finally, in the fourth study, all the experimental conditions consistently decreased their sympathovagal tone (decrease in LFnu), showing large effect sizes (May et al., 2019).

### Association Between HRV and EFs

As shown in Table 4, 83% of studies that reported improvements in EFs observed improvement in HRV, while 62% of studies that reported improvements in HRV observed improvements in EFs. However, analyzing the contingency table via a Fisher’s exact test revealed that there was no association between whether a study found improvements in HRV and whether it found improvements in EFs (*p* = 1).

**Table 4:** Cross-tabulation of HRV and EF outcomes following a biofeedback intervention

	EF improvements	No EF improvements	Total
HRV improvements	5	3	8
No HRV improvements	1	1	2
Total	6	4	10

## Discussion

The purpose of this systematic review was to examine and summarize the available literature on the effects of HRV-BF interventions on EFs and to address whether this literature suggests that HRV-BF interventions may indeed improve EFs. In addition, we aimed to investigate different factors that may affect whether an intervention leads to improvements in EFs. Findings show mixed results with regard to the efficacy of HRV-BF on EFs. Indeed, only nine out of the 16 studies included in this review (56%) saw improvements in one of the executive measures after the HRV-BF intervention. *Attention* was one of the domains that most often benefited from the intervention, with six out of eight studies finding positive changes. Effect sizes for within-group pre-test–post-test differences ranged from small (de Bruin et al., 2016) to medium (Groeneveld et al., 2019) to large (Jester et al., 2019). In a controlled study, de Bruin et al. (2016) found an advantage for the physical exercise group that performed better than the BF group, with a medium effect size. In the study of May et al. (2019), large effect sizes after HRV-BF were revealed, with the BF group performing significantly better than the high-intensity interval training group and a waiting list group. In the study of Rusciano et al. (2017), the BF group performed better than the control group with a large effect size, while Sutarto et al. (2013) showed a small to medium effect size of the intervention with no significant differences between the groups.

*Inhibition* was another domain that benefited from HRV-BF, with four out of six studies showing significant improvements. Effect sizes for pre-test–post-test controlled studies ranged from small-to-medium to large in two studies (Rusciano et al., 2017; Sutarto et al., 2013). The two remaining studies found a significant improvement but did not provide sufficient data for effect size computation (Ginsberg et al., 2010; Prinsloo et al., 2011).

Four studies assessed *working memory*. Two studies revealed improvements after the intervention but did not provide sufficient data to compute effect sizes (Ginsberg et al., 2010; Pop-Jordanova & Chakalaroska, 2008). For the two other studies, HRV-BF was not effective (Kenien, 2015; Raaijmakers et al., 2013). Finally, one study reported a small effect size of the HRV-BF intervention on *global executive functioning* (de Bruin et al., 2016). In contrast, none of the studies that assessed *cognitive flexibility* (four in total) found improvements. In the following sections, we will discuss the different factors that impact whether HRV-BF may (or may not) show benefits on EFs.

Regarding the population characteristics, our review shows that the target population plays a crucial role in the potential efficacy of the intervention. The majority of improvements (78%) occurred in studies that addressed patient populations or individuals that may present particular profiles: children and adults with ADHD, older adults with and without psychiatric symptoms, war veterans, stressed adults, and professional athletes. The remaining studies (22%) that reported significant improvements focused on student populations. Together, this suggests that HRV-BF is typically more beneficial for patient populations or individuals with particular profiles (e.g., individuals exposed to stressful environments and individuals with lower performance in baseline cognitive measures) than for the general population (i.e., students or healthy adults). This suggests that different mechanisms may lead to EF improvements after HRV-BF training. With regard to individuals exposed to stress, this can be understood in terms of the association between arousal and cognitive performance, which is well illustrated by the Yerkes–Dodson law (Teigen, 1994). According to this law, cognitive performance initially increases when physiological or mental arousal (e.g., stress) increases. However, this only continues until reaching an optimal level: when levels of arousal become too high, performance starts to decrease again. Thus, individuals with increased stress levels may have improved their cognitive functioning because the BF training taught them how to self-regulate and how to adjust their arousal to a more optimal level. Similarly, ADHD patients—another vulnerable population with high levels of arousal—also improved their attention, allowing them to shift from below-normative values before the intervention to in-norm values after the intervention (Groeneveld et al., 2019). In contrast, healthy populations that are less exposed to stressful environments may already have lower levels of arousal, which could explain the less effective impact of the intervention.

Furthermore, our results show that older patients also benefit from the HRV-BF intervention, regardless of their psychiatric conditions (Jester et al., 2019). Research in this domain typically suggests that psychiatric disorders (such as anxiety or depression) are negatively related to cognitive functioning. For example, Pacheco-Unguetti et al. (2010) suggest that trait anxiety is associated with lower cognitive control. In the same line, Fiske et al. (2009) showed that depression is related to cognitive impairment in older adults. Findings of Jester et al. (2019) suggest that HRV-BF intervention can be an effective method to improve attentional skills in older patients.

Regarding the intervention features, interestingly, the intensity of the treatment—in terms of the number of sessions, duration, and frequency—did not seem to impact the efficacy of the intervention. The studies reporting significant improvements largely varied in terms of intervention intensity: the total number of intervention sessions varied between one single session (Prinsloo et al., 2011) and 15 sessions (Rusciano et al., 2017). Similarly, among the studies which did not see positive changes following the intervention, the number of sessions varied from one (Lee & Finkelstein, 2015; Sherlin et al., 2010) to a not precise number of sessions spread over an academic semester (Bradley et al., 2010). Together, these results raise one relevant point: HRV-BF intervention can be effective whether it is delivered in small or high doses. However, the two studies which explored the effectiveness of a very short HRV-BF intervention on cognitive performance found contradictory findings. Prinsloo et al. (2011) showed some improvement in reaction time and accuracy following a single 10-min HRV-BF session. Lee and Finkelstein (2015), in contrast, found a deterioration of attention following a 10-min HRV-BF session. While Prinsloo et al. (2011) included a specific population (senior managers exposed to work-related stress), Lee and Finkelstein (2015) included healthy adults. This again suggests that populations with particular profiles may respond better and more rapidly even to a brief HRV-BF intervention than healthy individuals, making HRV-BF a promising treatment for context-specific punctual interventions.

Regarding the equipment, the studies included in the current review presented a significant heterogeneity in the type of technical devices that were used, with considerable differences in their possibilities and cost. Freeze-Framer/emWave and StressEraser were the most used devices (44% and 25%, respectively), followed by the NeXus-10 Mark II (12%) and, in equal percentages, ProComp Infiniti/BioGraph, Active Two, I-330C2, and BioPac/Elite (6% each). However, our findings suggest that the type of instrumentation does not impact the effectiveness of the intervention. In fact, Freeze-Framer/emWave and StressEraser (the most frequently used instruments) were used in successful and non-successful interventions.

## Limitations and Outlook

This systematic review faced several limitations. First, the included studies were largely heterogeneous regarding a series of conceptually relevant aspects: they importantly differed in terms of research designs, types of target populations, which measurements they used in order to assess EFs and how those measures were interpreted (e.g., similar tasks being interpreted as indicators of different executive facets between studies), and sample sizes. Second, studies largely differed regarding which effect size was reported (Cohen’s *d* vs. partial eta squared vs. no reported effect sizes) and on which comparison effect sizes were based on (pre–post comparison of the intervention group only vs. pre–post control group designs). In view of these large heterogeneities between studies, a major limitation of the current literature represents the challenge to properly compare (the extent of) improvements between studies and to pool effect sizes in order to draw firm quantitative conclusions on the effect of BF interventions. Thus, at this stage, we cannot conclude whether certain studies showed larger EF-specific benefits than others (e.g., whether improvements in a particular EF facet were larger in one compared to another study) nor establish which EF benefitted the most from the intervention (e.g., whether improvements in one EF are larger than those in another EF). Another key aspect in the lack of consistency of our results was the small sample sizes. This should be avoided when considering that insufficient sample sizes may have insufficient power and be the cause for the lack of significant findings for group differences that would actually exist. Further, even for those interventions that proved to be effective, the absence of a follow-up assessment does not allow us to know if the intervention effect would persist over time. It would be important to differentiate between short- and long-term effects in order to establish the real efficacy of HRV-BF as a potential intervention to enhance EFs. A final limitation is that half of the studies (eight out of 16) did not assess or report any HRV measures. Thus, it is difficult to assess whether intervention-related changes in HRV are associated to (changes in) EFs. Consequently, our review suggests that the available evidence in this research area is currently insufficient and that, at this stage, it would be inappropriate, or even misleading, to combine statistical results across individual studies (e.g., to conduct meta-analyses).

To overcome the different limitations presented above and to improve comparability across studies, future research should more consistently report effect sizes based on calculations for pre-test–post-test control group designs. More research in this field combined with more consistent reporting should allow performing meta-analyses that go beyond a descriptive review of the literature. This will further allow future research to apply similar and thus comparable intervention designs and protocols across studies that assess similar cognitive constructs. Future studies should also consider the inclusion of general populations, larger samples, and randomized controlled trials to detect consistent pre-test–post-test changes in EFs in the experimental group compared to one or more control groups. Finally, the association between improvements in HRV and improvements in EFs should be further investigated. Future studies—and meta-analyses in particular, as soon as those are possible—should examine in more detail whether the degree of change in HRV following a HRV-BF intervention directly translates into the amount of change in EFs.

## Conclusion

Taken together, the findings of this review suggest that HRV-BF interventions can benefit executive domains such as attention, inhibition, and working memory, but not cognitive flexibility. These benefits are independent of the intensity, the duration, or the type of technical equipment used for the intervention. However, at this stage, it is unclear to which extent this technique can improve such domains and whether one domain benefits to a larger degree than the others. Further, today, it also remains unclear how improvements in HRV relate to improvements in EFs (whether and how the degree of change in HRV following a BF intervention is associated to the degree of change in EFs).

In terms of target populations, patients of different ages, older adults, and adults exposed to stressful working conditions seem to particularly benefit from HRV-BF interventions. Thus, HRV-BF could, for example, be helpful in the context of clinical rehabilitation programs that aim to treat ADHD symptoms in children and adults. Also, HRV-BF could be applied as an intervention in the workplace to help stressed employees strengthen executive functioning. HRV-BF could also be implemented in assisted living communities as an effective method to counteract cognitive decline and support the independence of vulnerable older adults by achieving or maintaining an optimal level of executive functioning. Finally, the important heterogeneity of the studies included in this systematic review also underlines the need for more standardized research designs, for more consistent reporting, and generally, for more research in this field.

## Supplementary Information

Below is the link to the electronic supplementary material.

Supplementary file1 (DOC 67 KB)

## Data Availability

Not applicable.

## Footnotes

1 NN intervals: interbeat intervals after artifact removal.

2 RR interval: interbeat intervals between all successive heartbeats.
